# CD56bright Natural Killer Cells: A Possible Biomarker of Different Treatments in Multiple Sclerosis

**DOI:** 10.3390/jcm9051450

**Published:** 2020-05-13

**Authors:** Alice Laroni, Antonio Uccelli

**Affiliations:** 1 Department of Neuroscience, Rehabilitation, Ophthalmology, Genetics, Maternal and Child Health and Center of Excellence for Biomedical Research, University of Genova, 16132 Genova, Italy; auccelli@neurologia.unige.it; 2 IRCCS Ospedale Policlinico San Martino, 16132 Genova, Italy

**Keywords:** multiple sclerosis, natural killer cells, CD56^bright^ NK cells, NK regulatory cells, biomarker, disease-modifying treatments, innate immunity

## Abstract

Multiple sclerosis (MS) is an immune-mediated disease of the central nervous system, which leads, in many cases, to irreversible disability. More than 15 disease-modifying treatments (DMTs) are available for the treatment of MS. Clinical activity or activity at magnetic resonance imaging (MRI) are now used to assess the efficacy of DMTs, but are negative prognostic factors per se. Therefore, a biomarker permitting us to identify patients who respond to treatment before they develop clinical/radiological signs of MS activity would be of high importance. The number of circulating CD56^bright^ natural killer (NK) cells may be such a biomarker. CD56^bright^ NK cells are a regulatory immune population belonging to the innate immune system. The number of CD56^bright^ NK cells increases upon treatment with interferon-beta, alemtuzumab, dimethyl fumarate, after autologous hematopoietic stem cell transplantation, and is higher in those who respond to fingolimod. In some cases, an increased number of CD56^bright^ NK cells is associated with an increase in their regulatory function. In the current review, we will evaluate the known effect on CD56^bright^ NK cells of DMTs for MS, and will discuss their possible role as a biomarker for treatment response in MS.

## 1. Introduction

Multiple sclerosis (MS) is an immune-mediated disease of the central nervous system (CNS), which may lead to irreversible disability [1]. In most cases, it begins with a relapsing course, characterized by bouts of inflammatory cells going from peripheral blood to the CNS, causing new lesions in the brain and spinal cord, some of them being associated with clinical symptoms (relapses) [1].

The therapeutic arsenal for relapsing MS counts more than 15 disease-modifying treatments (DMTs), all of them affecting the immune function at different levels [2,3]. Clinical trials inform about the efficacy of each drug in the overall population, but it is still impossible to predict whether single subjects will respond to a specific treatment or not. The definition of responders itself is not univocal: the more treatments are approved, the higher the bar of expectation is raised, with the ultimate goal of achieving “no evidence of disease activity” (NEDA) as evaluated by clinical parameters (no relapses, no progression of disability) and magnetic resonance imaging (MRI: no new or active lesions, no atrophy) [4]. However, when a patient starts treatment, there are no biological markers to predict disease reactivation before it becomes visible at MRI or at the clinical level, i.e., when inflammation has already mediated some damage to the CNS tissue [5].

From a pathophysiological point of view, immune cells belonging to the adaptive immune system (T and B lymphocytes) are the main players in the initiation of MS after their activation in peripheral organs and their subsequent passage through the barriers that divide them from the CNS [1,6]. What causes their activation has not been univocally defined; however, a dysfunction in regulatory immune cells in MS has been shown both for the adaptive and the innate components [7].

Among innate cells, a subset of natural killer (NK) cells, the CD56^bright^ NK cell subset, has emerged as having a regulatory function, which is impaired in MS. Moreover, and interestingly, independent studies evaluating the immune effects of different DMTs have shown, in many cases, an increase in the number of CD56^bright^ NK cells upon treatment. In this review, we will summarize what is known about the role of CD56^bright^ NK cells in MS and the effects of DMTs on such regulatory innate cell subsets, focusing on treatment with known effects on NK cells.

## 2. CD56^bright^ NK Cells: A Regulatory Immune Subset

NK cells are a part of innate lymphoid cells within the innate immune system [8]. NK cells are cytotoxic towards cells infected by viruses and cancer cells and are able to select their targets through receptors which recognize self-molecules (mainly human leukocyte antigen—HLA—class I molecules), inhibiting their activation, and other receptors which bind to ligands expressed by stressed cells, to pathogen-related ligands, or to unknown ligands, mediating their activation [9].

NK cells are not a homogeneous population, but include different subsets with specific functions. In humans, NK cells in the peripheral blood can be divided into two main subsets: CD56^dim^ NK cells, more abundant and highly cytotoxic against cells infected by viruses or cancer cells, and CD56^bright^ NK cells [10]. The latter subset is phenotypically and functionally different from CD56^dim^ NK cells in many ways. From a phenotypic point of view, CD56^bright^ NK cells have a specific array of surface receptors: their main inhibitory receptor is the NK group 2 member A (NKG2A), while they usually lack the killer immunoglobulin receptors (KIR) which are expressed by CD56^dim^ NK cells [11]. Moreover, only a minority of them express the Fc-gamma receptor, CD16, which is expressed by all CD56^dim^ NK cells [11].

Developmentally, CD56^bright^ NK cells are hypothesized to descend from the common lymphoid precursor, representing an earlier differentiation stage of NK cells, and to give rise to the CD56^dim^ NK cells [12]. This “linear” hypothesis of differentiation is corroborated by several observations, including increased length of telomeres of CD56^bright^ NK cells compared to CD56^dim^ NK cells [12], and prevalence of CD56^bright^ NK cells in peripheral blood in the early phases after hematopoietic stem cell transplantation, as summarized by [13].

However, other studies challenge such a model, including an important one in rhesus macaques, transplanted with human hematopoietic cells, which suggests that different subsets of NK cells are clonally unrelated [14]. Different hypotheses on the differentiation of NK cell subsets are reviewed in [15].

From a functional point of view, CD56^bright^ NK cells have been described as “immunoregulatory” since their first characterization, due to their lower capability of killing standard targets (i.e., cancer cell lines lacking HLA class I molecules), and higher production of cytokines, compared to CD56^dim^ NK cells [16]. However, later studies have shown that, upon appropriate stimulus, CD56^bright^ NK cells are indeed capable of cytotoxicity toward certain targets, and particularly towards healthy, autologous T cells.

Indeed, CD56^bright^ NK cells and T cells have bilateral interactions. They co-localize in secondary lymphoid organs, and particularly in T-cell areas of the lymph nodes (LNs), where interleukin (IL)-2 produced by T cells activates CD56^bright^ NK cells after binding to its high-affinity receptor on their surface [17]. CD56^bright^ NK cells produce cytokines, including interferon (IFN)-gamma and IL-10, which can shape T-cell responses [16]. Perhaps more importantly, CD56^bright^ NK cells can suppress T-cell responses through many mechanisms. We have shown that when CD56^bright^ NK cells are exposed to a cytokine produced by antigen-presenting cells (APCs), IL-27, they can suppress the proliferation of autologous T-cells [18]. Morandi et al. described another way through which CD56^bright^ NK cells suppress T-cell proliferation, which involves the release of adenosine [19]. Nielsen et al. described the cytotoxicity of CD56^bright^ NK cells towards autologous T-cells after the engagement of activating receptors [20]. We have shown that upon stimulus with proinflammatory cytokines produced by APCs, Ex Vivo isolated CD56^bright^ NK cells become able to kill activated, autologous CD4+ T-cells through a contact-dependent mechanism that involves the activating receptor natural killer cell P46-related protein (NKp46) on CD56^bright^ NK cells [21]. In this context, cytotoxicity results in suppression of T-cell proliferation. Similarly, Gross et al. have shown that NK cells kill autologous T-cells through the engagement of the activating receptor DNAX accessory molecule 1 (DNAM-1) [22]. Darlington et al. have reported that total NK cells modulate the polarization of T-cells towards T helper 17 (Th17) cells, initially increasing the production of IL-17 by T-cells, but then killing Th17 cells through a mechanism involving the activating receptor Natural Killer Group 2D (NKG2D). [23].

CD56^dim^ NK cells share only some regulatory features with CD56^bright^ NK cells. Compared to CD56^bright^ NK cells, CD56^dim^ NK cells do not suppress T-cell proliferation upon stimulus [21]; considering cytotoxicity, one study showed that CD56^bright^ killed only activated autologous T-cells and did not target resting T-cells, whereas CD56^dim^ killed resting T-cells as well; the same study showed that mechanism of killing is different among the two subsets [24]; another study showed that different stimuli are required by CD56^bright^ and CD56^dim^ NK cells in order to become able to kill T-cells [20].

Altogether, these studies demonstrate consistently that different proinflammatory stimuli activate different pathways, all leading to the cytotoxicity of NK cells, and particularly CD56^bright^ NK cells, towards autologous, activated T-cells, and/or to NK cell-mediated suppression of proliferation of T-cells [25]. Since it is likely that such function occurs physiologically in secondary lymphoid organs, we can hypothesize that it has a role in preventing the excess of response of T-cells to proinflammatory stimuli upon activation by APCs.

## 3. NK Cells in Untreated MS Patients and in Patients Undergoing Disease-Modifying Treatments

### 3.1. NK Cells in Untreated MS Patients

In untreated MS, the number of CD56^bright^ NK cells is the same as in healthy subjects [21]. However, their regulatory function is impaired, as shown by different groups, including ours. We have shown that CD56^bright^ NK cells from untreated MS patients are unable to suppress the proliferation of activated autologous CD4+ T cells. Such impairment was associated with an upregulation of T-cells of HLA-E, which is a ligand to the inhibitory receptor NKG2A expressed on CD56^bright^ NK cells. Therefore, we hypothesize that CD4+ T-cells from untreated MS patients, through the upregulation of HLA-E, inhibit the cytotoxicity of CD56^bright^ NK cells [21]. Gross et al. have reported a similar impairment in the cytotoxicity of NK cells towards autologous activated T-cells in MS. More specifically, they observed that untreated MS patients have both a decrease in the expression of the activating receptor DNAM-1 on NK cells and in the expression of its ligand on T-cells, CD155 [22]. Another study reported a decreased cytotoxicity of NK cells from MS patients towards target cell lines, and lower production of some cytokines, including IFN-gamma [26].

The antiviral function of CD56^dim^ NK cells may be important in controlling MS. CD56^dim^ NK cells are involved in the immune response against infection by cytomegalovirus (CMV), and such an infection is associated with a lower risk of developing MS. A recent study showed that NK cells from patients with progressive MS, and particularly, primary progressive MS, have lower effector function towards target cells; the authors speculate that this might contribute to lower capability of killing viruses such as the Epstein-Barr virus, which may be involved in MS pathogenesis [27]. Others hypothesize that CD56^dim^ NK cells could be detrimental: increased numbers of CD56^dim^ NK cells expressing perforin have been found in patients with secondary progressive MS [28].

It still has to be proven whether such impairment contributes to causing MS or is the result of other causal factors. Nevertheless, it is interesting to note that an increase in the number of CD56^bright^ NK cells and/or enhancement of their immunomodulatory function has been observed in patients treated with DMTs.

Finally, some authors have found an association between NK cell phenotype and disease status in MS: more specifically, NK cells from patients in remission express higher levels of FS-7-associated surface antigen (Fas) and suppress autoimmune T-cell responses, and loss of such Fas-high NK cells is associated with relapse activity [29,30].

### 3.2. NK Cells in Patients Undergoing Disease-Modifying Treatments for MS 

#### 3.2.1. Interferon-Beta

Long before the impairment in the regulatory function of NK cells in MS was described, one report was published suggesting that different formulations of IFN-beta (IFN-b), the first approved DMT for MS, increase the number of CD56^bright^ NK cells [31]. IFN-b molecules are type 1 IFN cytokines with a pleiotropic mechanism of action that involves modulation of the expression of several genes. This translates into the modulation of many functions of immune cells, including antigen presentation and regulatory features [32]. Type 1 IFN has a direct effect on NK cells, increasing their cytotoxicity and anti-tumor function [33]; moreover, IFN-alpha, another type 1 IFN molecule, has been shown to specifically expand CD56^bright^ NK cells and to increase their expression of activating receptors, in the context of hepatitis B treatment [34]. All IFN-b formulations are injectables and have a similar impact on MS, reducing relapses and progression by about 30% compared to placebo [35]. In two studies on small groups of patients treated with IFN-b, the authors found that the proportion of whole NK cells among peripheral mononuclear cells decreased, but CD56^bright^ NK cells ratio to CD56^dim^ NK cells increased [31,36]. Increased numbers of CD56^bright^ NK cells in patients treated with IFN-b have been described in another study, both cross-sectionally (compared to untreated) and longitudinally (at 24 months after treatment compared to before treatment start) [37].

The mechanism through which type 1 IFN expand NK cells is debated, with one study suggesting that it is an indirect mechanism, mediated by increased production of cytokines by monocytes [38].

#### 3.2.2. Daclizumab

Subsequently, an increase in CD56^bright^ NK cells upon treatment with the anti-CD25 monoclonal antibody daclizumab was reported and has been regarded as one of the main mechanisms of action of daclizumab in MS. Several studies, led by the seminal one of Bielekova et al., demonstrated that daclizumab enhanced the cytotoxicity of CD56^bright^ NK cells and reverted their impaired regulatory function (see, for instance, [24,39]). The mechanisms through which CD56^bright^ NK cells killed T-cells upon daclizumab treatment included the transfer of granzyme K, the expression of which was induced by treatment [39], and reversal of the decreased expression of CD155 on T-cells [22]. The mechanisms of induction of CD56^bright^ NK cells upon daclizumab treatment likely depend on increased availability of IL-2, due to blockade of the high-affinity IL-2 receptor on T-cells [40,41].

Unfortunately, shortly after daclizumab was authorized as a DMTs for MS, 12 cases of autoimmune encephalitis in treated patients led to its withdrawal from the market [42]. The reason for such adverse events has not yet been elucidated, but might possibly be related to the decrease in the number of T regulatory cells caused by daclizumab [42].

Interestingly, a relative or absolute increase in the number of CD56^bright^ NK cells was then reported with other DMTs for MS.

#### 3.2.3. Dimethyl Fumarate

Dimethyl fumarate (DMF) is a fumaric acid ester approved as an oral compound for the treatment of MS. Treatment with DMF decreased relapse rate in two Phase 3 placebo-controlled clinical trials and decreased disability progression in one of the two trials [43,44]. DMF and its active metabolite monomethyl fumarate through activation of receptors, such as the hydroxycarboxylic acid receptor 2, target intracellular pathways, including the nuclear factor-kappa B pathway, decreasing the activation of immune and glial cells [45].

In subjects treated with DMF, a study by Medina et al. reported an increased number of CD56^bright^ NK cells at six months of treatment in the overall population, and particularly in those achieving NEDA in the first year of follow up [46]. Subsequently, the group of Peter Calabresi confirmed the increase of CD56^bright^ NK cells upon DMF treatment, with an inverse correlation between the number of CD56^bright^ NK cells and the number of CD8+ T cells. The same study showed that In-Vitro treatment of NK cells with the drug, or with monomethyl fumarate, increases the cytotoxicity of NK cells towards autologous T-cells [47]. Increased numbers of CD56^bright^ NK cells or total NK cells upon DMF treatment has been confirmed by other groups [48,49]. It is still unknown how DMF induces an increase in the number of CD56^bright^ NK cells; we can speculate that a decrease in T-cell numbers, (possibly specifically CD8+ T-cells) caused by DMF, increases the availability of cytokines and triggers the expansion of CD56^bright^ NK cells.

#### 3.2.4. Fingolimod

In the scenario of fingolimod treatment, results are more complex. Fingolimod is a functional antagonist of the sphingosine-1 receptors, which are involved in the egress of lymphocytes from secondary lymphoid organs (SLOs). Treatment with fingolimod causes lymphopenia of variable degrees due to the retention of lymphocytes in SLOs [50]. Treatment with fingolimod reduces relapse rate and progression of disability in relapsing–remitting MS compared to placebo and to IFN-b [51,52].

One study reported a relative increase of NK cells over other immune cells in blood and cerebrospinal fluid of a small group of patients treated with fingolimod, likely due to relative sparing of NK cells compared to B- and T-cell subsets [53]. In three cohorts of patients treated with fingolimod, the increased proportion of circulating NK cells was found to be associated with a decreased number of CD56^bright^ NK cells, consistently with their expression of the chemokine receptor C-C chemokine receptor type 7 (CCR-7), which mediated their migration to the LNs [54,55,56]. However, a lower decrease in CD56^bright^ NK cells during treatment with fingolimod was found in responder patients, as discussed further in the paragraph detailing the clinical impact of the expansion of NK cells upon treatment [57].

While one study showed no effect of fingolimod on the transcriptomic signature of circulating NK cells in treated subjects [58], another In-Vitro study reported that the culture of NK cells in the presence of fingolimod induces higher expression of natural cytotoxicity receptors and increases cytotoxicity toward target cell lines [59]. It would be of interest to assess whether this is associated with increased cytotoxicity of NK cells towards autologous T-cells.

#### 3.2.5. Alemtuzumab

Alemtuzumab is an anti-CD52 monoclonal antibody approved for the treatment of relapsing MS. Alemtuzumab binds to the CD52 antigen expressed by adaptive immune cells and, in lower amounts, by innate immune cells [60]. The number of T- and B-lymphocytes of treated patients decreases immediately after treatment, mainly due to antibody-dependent cellular cytotoxicity (ADCC) and later reconstitutes [61]. Alemtuzumab is administered intravenously in a five-day course, followed by a three-day course after one year, inducing a decrease in the relapse rate of about 50% compared to IFN-b treatment. The effect of treatment is long-lasting in about 70% of treated patients [35]. About one-third of MS patients treated with alemtuzumab develop secondary autoimmunity, which demonstrates that alemtuzumab has profound effects on immune mechanisms; however, the mechanisms leading to secondary autoimmunity are currently unknown [62]. Gross et al. found that the first course of treatment with alemtuzumab leads to an increased number of CD56^bright^ NK cells at six months. Cytotoxicity of CD56^bright^ NK cells from these patients, as evaluated towards an HLA-deficient target cell line, was unchanged [60]. It is unknown why alemtuzumab increases CD56^bright^ NK cells, and whether such an increase has a clinical meaning. We may hypothesize that the cytokine storm occurring immediately after the administration of alemtuzumab triggers the expansion of CD56^bright^ NK cells [63]. Another hypothesis is that the profound T-cell decrease caused by alemtuzumab is involved in the expansion of CD56^bright^ NK cells, similarly to what is discussed below in the context of hematopoietic stem cell transplantation. Importantly, NK cells and particularly CD56^dim^ NK cells mediate ADCC upon treatment with alemtuzumab, through the engagement of the CD16 receptor with the fragment crystallizable region (Fc region) of alemtuzumab.

#### 3.2.6. Autologous Hematopoietic Stem Cell Transplantation

Autologous hematopoietic stem cell transplantation (AHSCT) is a treatment for very active and refractory relapsing MS. ASHCT has excellent long-term outcomes, despite some treatment-related mortality, which has significantly lowered in the last years [64]. One study from Peter Darlington et al., within the Canadian MS/Bone Marrow Transplantation Study Group, compared the kinetics of NK cell and CD4+ T-cell reconstitution after AHSCT [23]. The authors reported a marked increase of NK cell/CD4+ T-cell ratio at three months compared to baseline. Both CD56^bright^ NK cells and CD56^dim^ NK cells increased after treatment, albeit to a greater extent for CD56^bright^ NK cells. Moreover, in post-AHSCT samples, the depletion of NK cells increased Th17 cell polarization. The authors concluded that expanded NK cells contribute to suppressing Th17 cell responses after AHSCT and contribute to its extraordinary effectiveness in treating MS [23]. Expansion of CD56^bright^ NK cells after hematopoietic stem cell transplantation in disease settings different from MS is a well-known phenomenon, which has been considered as proof for CD56^bright^ NK cells representing an earlier stage of differentiation, as discussed before [65]. However, expansion of CD56^bright^ cells might also represent a specific compensatory mechanism in the absence of CD4+ T cells, as shown by studies that found an inverse correlation between the number of CD4+ T-cells and the number of CD56^bright^ NK cells after hematopoietic stem cell transplantation. Such studies suggest that decreased competition for cytokines by CD4+ T-cells, or undergoing infectious events, might be the trigger for expansion of CD56^bright^ NK cells [66,67].

Altogether, these data suggest that CD56^bright^ NK cells may be a biomarker of treatment efficacy in MS.

Table 1 summarizes the findings of the studies on the effect of DMTs on NK cells and NK cell subsets.

### 3.3. Clinical Impact of Expansion of NK Cells Upon Treatment

In order to assess whether the expansion of CD56**^bright^** NK cells is an epiphenomenon of treatments, or rather is involved in mediating their effects, some studies compared the effect of DMTs on NK cells in patients who responded to treatment and in those who did not. In two different studies, involving patients treated with IFN-b and with DMF, only responders had a significant increase in CD56^bright^ NK cells [37,46]. This observation may suggest that increased numbers of NK cells with immunoregulatory features are beneficial in controlling MS disease activity; however, another alternative explanation may be that the low number of non-responders, in both studies, may have limited the possibility to reach statistical significance. In patients treated with daclizumab, there was a clear correlation between the increase in CD56^bright^ NK cells and the decrease in the number of active lesions at MRI [24]. In another study in daclizumab-treated patients, numbers of CD56^bright^ NK cells were inversely correlated with the number of new or newly enlarging lesions at MRI. The authors observed a similar trend of inverse correlation between numbers of CD56^bright^ NK cells and relapses. Since lower disease activity compared to placebo was also observed in patients without expansion of CD56^bright^ NK cells, the authors concluded that CD56^bright^ NK cells are responsible to some, but not the whole, effect of daclizumab [68]. An elegant study by Moreno-Torres et al., aimed at finding biomarkers of response to fingolimod in 40 treated patients, found that patients who achieved NEDA status at one year had higher percentages of CD56^bright^ NK cells at baseline (i.e., before starting fingolimod) and that, upon treatment, CD56^bright^ NK cells decreased less in these patients compared to non-responders [59].

Finally, one study in MS patients treated with different drugs (natalizumab, fingolimod, glatiramer acetate, or IFN-b) reported a correlation between increased CD56^bright^ NK cells and no evidence of disease activity at MRI. A relative increase in CD56^bright^ NK cells compared to CD56^dim^ NK cells and, at least in patients treated with fingolimod, compared to T-cells, rather than an increase in absolute numbers, was associated with treatment response [69].

All these data suggest that different treatments for MS enhance the numbers, and in some cases (DMF, daclizumab, AHSCT), the regulatory function of CD56^bright^ NK cells. Such effect is involved in mediating at least some of their clinical efficacy, possibly through modulation of the function of adaptive cells involved in the attack against the myelin (CD4+ and CD8+ T-cells).

## 4. Discussion and Conclusions

**Figure 1:**
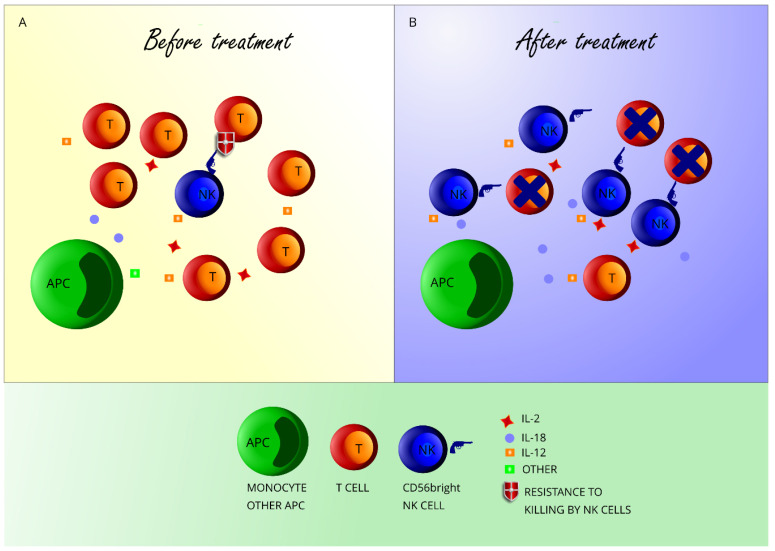
CD56^bright^ NK cell expansion after DMT in multiple sclerosis (MS). Panel A: Before treatment, antigen-presenting cells (APC) secrete cytokines that activate T-cells and CD56^bright^ NK cells. T-cells outnumber CD56^bright^ NK cells and are resistant to killing by CD56^bright^ NK cells through various mechanisms. Panel B: DMTs decrease T-cell numbers, leading to the increased availability of cytokines secreted by APC (such as IL-18 and IL-12) and by T-cells themselves (IL-2) to CD56^bright^ NK cells. Some treatments may restore the killing of T-cells by CD56^bright^ NK cells. Some treatments might also induce higher secretion of cytokines by APC.

The role of adaptive immune cells in initiating the inflammatory cascade in MS is crucial. However, many clues point to NK cells, a lymphocyte population belonging to the innate compartment, as another important immune population in MS [70]. First of all, a failure in CD56^bright^ NK cell-mediated control of T-cell responses exists in untreated subjects with MS; it is not known whether such impairment is concurring to causing MS, and further research, for instance in at-risk subjects, may help in elucidating this issue. Secondly, different treatments that ameliorate the MS disease course enhance the absolute or relative number of CD56^bright^ NK cells in the peripheral blood. It is important to point to the fact that the number of CD56^bright^ NK cells in untreated MS subjects is not decreased; however, an increase in CD56^bright^ NK cells appears to be a very consistent effect of many therapies. Such observation may suggest that CD56^bright^ NK cells are an important cell subset for controlling adaptive immune responses. We hypothesize that DMTs affect CD56^bright^ NK cells through different mechanisms. Effect of DMTs on CD56^bright^ NK cells may depend on increased availability of cytokines in the context of decreased CD4+ and/or CD8+ T-cell numbers (alemtuzumab, AHSCT, DMF, fingolimod), or on the increased secretion of cytokines by target cells/direct stimulus on NK cells (IFN-b; Figure 1).

As discussed before, and summarized in Table 1, the effect of treatment on CD56^dim^ NK cells is variable and not as consistent as observed with CD56^bright^ NK cells. CD56^dim^ NK cells proliferate less compared to CD56^bright^ NK cells in the presence of cytokines [16]; thus, increased availability of cytokines due to T-cell loss would specifically expand the CD56^bright^ NK cell subset. Given the higher immunoregulatory function of CD56^bright^ NK cells, this would translate into a modulation of adaptive responses. Nevertheless, we cannot exclude that increased numbers of CD56^dim^ NK cells upon specific treatments (AHSCT, and possibly fingolimod and alemtuzumab) play a role in the mechanism of action of such drugs. Moreover, the proportion and function of CD56^dim^ NK cells before treatment start may influence the biological effect, and thus the clinical efficacy of drugs that rely on them for their function, such as alemtuzumab. Indeed, there are inter-individual differences in the depletion and subsequent repopulation of lymphocytes after alemtuzumab treatment [71], and it would be interesting to investigate whether baseline numbers of CD56^dim^ NK cells influence such differences.

In conclusion, we propose that CD56^bright^ NK cells should be studied as a possible biomarker of DMTs efficacy in MS. Having a biomarker of treatment efficacy from a blood sample would be of the highest importance in MS. This would be particularly relevant if it were demonstrated that failure in the increase in CD56^bright^ NK cells is associated with later MRI activity and/or clinical relapse. In this case, patients without an increase of CD56^bright^ NK cells upon DMTs could be evaluated for a possible change of treatment before clinical/radiological activity occurs.

Several open questions still exist: for instance, whether there is one cutoff value for CD56^bright^ NK cell number that could be implemented in the clinical practice, or whether different treatments may enhance CD56^bright^ NK cells differently (as data with fingolimod-treated patients suggest) and therefore a treatment-specific CD56^bright^ NK cell cutoff value could be identified. Thirdly, an important question that is still to be answered is how such an increase in the number of CD56^bright^ NK cells affects adaptive immune responses in MS. Finally, we should clarify if and how changes in CD56^bright^ NK cells in the peripheral blood reflect changes in CD56^bright^ NK cells within the CNS, as data from daclizumab studies suggest [72]. Answering these questions may lead us to have CD56^bright^ NK cells as a novel, easy to measure, and reliable marker of treatment efficacy in MS.

## Figures and Tables

**Table 1:** Effect of disease-modifying treatments (DMTs) on natural killer (NK) cells and NK cells subsets. Each row represents a study. Abbreviations: absolute (Abs); unchanged (=); number (#); Peripheral Blood Mononuclear Cells (PBMCs); Interferon-beta (IFN-b); Dimethyl fumarate (DMF); Autologous hematopoietic stem cell transplantation (AHSCT).

DMT	Effect of DMT on Circulating Total NK Cells	Effect of DMT on Circulating CD56^bright^ NK Cells	Effect of DMT on Circulating CD56^dim^ NK Cells	# of Enrolled Subjects	Reference
**IFN-b**	↓ % NK of PBMCs at 12 months	↑ % CD56^bright^ of PBMCs at 3 and 12 months ↑ % CD56^bright^ of NK cells at 12 months	↓ % CD56^dim^ of PBMCs at 12 months	11	[31]
	Unchanged	↑ % CD56^bright^ of PBMCs at 12 months		11	[36]
		↑ % CD56^bright^ of NK cells at 24 months ↑ % CD56^bright of NK^ compared to untreated ↑Abs No compared to untreated	↓ % CD56^dim^ of NK compared to untreated ↓ Abs No compared to untreated	25 (longitudinal) 27 (cross-sectional)	[37]
**DMF**	= % NK of PBMCs at 6 months	↑ % CD56^bright^ of PBMCs at 6 months		64	[46]
	= % NK of PBMCs at 6 months	↑ % CD56^bright^ of NK cells at 6 months		18	[47]
		↑ Abs number at 12 months	↓ Abs number at 12 months	12	[48]
	↑ Abs number at 12 and 24 months				[49]
**Fingolimod**	↑ NK cells in treated vs. untreated			20 untreated, 12 treated	[53]
	↑ % NK cells of PBMCs in treated vs. untreated	↓ % CD56^bright^ of PBMCs in treated vs. untreated		NK: 5 untreated, 8 treated CD56^bright^: 8 untreated, 10 treated	[54]
		↓ Abs number at 6 h	= Abs number at 6 h	8	[55]
	= Abs number at 24 months	↑ Abs number at 24 months ↓ % CD56^bright^ of PBMCs at 24 months	= Abs number at 24 months ↑ % CD56^dim^ of PBMCs at 24 months	36	[56]
		↑ % CD56^bright^ of NK at 6 months (responders)		40	[57]
**Alemtuzumab**		↑ % CD56^bright^ of PBMCs at 6 months ↑ Abs number at 6 monthsnkg2d	=% CD56^dim^ of PBMCs at 6 months = Abs number at 6 months	12	[60]
**AHSCT**	↑ Abs Number at 12 and 21 months compared to 3 weeks after treatment ↑ % NK of PBMCs at 3–6–9–12–15–18 months	↑ % CD56^bright^ of PBMCs at 3-6-9-12 months ↑ % CD56^bright^ of NK cells at 3 and 6 months	↑ % CD56^dim^ of PBMCs at 3, 6, 9, 12, 15, 18 months	7	[23]
